# Potential of mealworms used in polyhydroxyalkanoate/bioplastic recovery as red hybrid tilapia (*Oreochromis* sp.) feed ingredient

**DOI:** 10.1038/s41598-022-13429-1

**Published:** 2022-06-10

**Authors:** Idris Zainab-L, Wing‐Keong Ng, Kumar Sudesh

**Affiliations:** 1 School of Biological Sciences, Universiti Sains Malaysia, 11800 Penang, Malaysia; 2 Asian Aquafeeds Services, Lavinia, Taman Sri Nibong, 11900 Penang, Malaysia

**Keywords:** Biotechnology, Microbiology

## Abstract

Polyhydroxyalkanoates (PHAs) are bio-based polymers produced in bacterial cells to replace some petrochemical plastics. It has always been a challenge to commercialise PHA due in part to the costly recovery processes of the PHA granules from the bacterial cells. The biological approach of using mealworms, *Tenebrio molitor,* for the recovery of PHA from the bacterial cells is a newly established method that is at the scale-up stage. On the other hand, the aquaculture feed industry needs a low-cost mealworm meal as a protein source. We aimed at studying the nutritional value of the mealworms (which are by-products) used for the poly(3-hydroxybutyrate) (PHB) (the most common type of PHA) recovery from the bacterial and examining the effect of the mealworms on the growth performance, and feed utilization efficiency of red hybrid tilapia (*Oreochromis* sp.). The cells were fed to the mealworms to digest the proteinaceous cellular materials and excrete the PHB granules in the form of fecal pellets. The resulting mealworms were used as fishmeal replacement to formulate five isonitrogenous (35% crude protein) and isolipidic (8% lipid) diets at mealworm meal (MwM) inclusion levels of 0% (MwM0/control diet), 25% (MwM25), 50% (MwM50), 75% (MwM75) or 100% (MwM100). The results showed good nutritive value mealworms [high protein (75%), low-lipid (10%)] and up to 75% MwM inclusion diet was good in supplying satisfactory nutrients and energy to the red hybrid tilapia. This approach is beneficial in a way that minimal cost was involved in recovering kilograms of PHB and the proteins, lipids, and minerals from the bacterial cells do not end up as wastes but in turn, are used as nutrition by the larvae.

## Introduction

Environmental pollution from the utilisation of petroleum-based plastics has triggered the search for alternative bio-based materials for various applications^1,2^. Intensive research has been conducted in the field of bio-based materials like polylactic acid (PLA) and polyhydroxyalkanoates (PHA). PHA is a family of microbial polyesters that is completely biodegradable and possesses the thermal and mechanical properties of some commonly used conventional plastics. The environmentally friendly PHA is an intracellular product synthesized by Gram-positive, Gram-negative bacteria, and Archaebacteria via the fermentative process on a wide range of substrates such as vegetable oils, sugars, fatty acids, and glycerol^3,4^. Though PHA is a desirable material, the high cost of carbon substrate and downstream recovery has become the major restriction for its commercialization. The downstream process (pretreatment, extraction, and purification) accounts for more than 30% of the PHA production cost^5^. Efforts have been made to recover and purify PHA through simplified recovery methods^6^. The conventional hazardous solvent and an expensive enzymatic digestion method are unfortunately not feasible for large-scale applications^7^.

The quest for a sustainable and greener method led to the discovery of the biological method of PHA recovery from freeze-dried bacterial cells (*Cupriavidus necator* H16) using Sprague Dawley rat and mealworms (the larvae of darkling beetles, *Tenebrio molitor*) by our group^8–11^. The concept of biological recovery is a breakthrough for the field of PHA as it does not involve the use of hazardous solvents such as chloroform and methanol. The freeze-dried cells containing the PHA were directly fed to the mealworms to digest the proteinaceous cellular materials and excrete the PHA granules in the form of fecal pellets at no or minimal cost. Furthermore, the proteins, lipids, and minerals from the bacterial cells do not end up as wastes but in turn, are used as nutrition by the mealworm larvae. *C. necator* H16 (formerly known as *Hydrogenomonas euthropha*) contain protein of high biological value comparable to casein, and it was well-tolerated by rats^11,12^, although the indigestibility of Poly(3-Hydroxybutyrate) (PHB) (a type of PHA) in the gastrointestinal tract (GIT) of animals was earlier reported by Waslien and Calloway^13^. The animals utilized the protein and other cellular components (usually discarded as a by-product) as nutrients while the intracellular PHB granules were excreted in the form of white faecal pellets.

The preference for insects over animals for the biological recovery of PHA was due to their worldwide distribution, ease of rearing, their requirement for less space, water, and simple management to transform low-value substrates/organic matter into body biomass and value-added products^14,15^. In addition to mealworms, a trial experiment with other insects showed that cricket, cockroach, and superworm can readily consume the freeze-dried bacterial cells and excreted the white faecal pellets^16^. Mealworms were chosen for their ease of cultivation under dry conditions and longer larval periods (2–3 months). During the larval stage, mealworms can absorb a substantial amount of nutrients from the bacterial cells and recover a large quantity of PHA granules. The biological method of PHA recovery using mealworms^16,17^ fits the concept of industrial symbiosis whereby the bioplastic industry provides the freeze-dried cells to the mealworm farm at little or no cost and the farm returns the faecal pellets containing the PHA to the bioplastic industry. The mealworm farm saves on the cost of feed at this stage and can reduce the selling price of mealworms.

Mealworms are traditionally produced on oats, flour, and wheat bran fortified with yeast as feed for household pets or zoo animals, fishing bait, and even for human consumption^18^. Mealworms are rich in protein, lipids, minerals, vitamins^19,20^, and a substantial amount of certain minerals^21,22^. The nutritional composition of mealworms varies by their diet and stages of development. For instance, some researchers reported as high as 68% and 76% protein content from mealworms fed on wheat bran and wheat straw (*Triticum aestivum* L.) diet respectively^23^, while others reported only 20% protein content^24,25^. In the case of the black soldier fly fed with coconut endosperm waste, the reported protein and lipid contents were 50% and 40%, respectively^15^. Recently, insects have been gaining attention as a potential source of protein for both human food and animal feed. As part of the natural diet of freshwater and marine fish with no known anti-nutritional factors^26,27^, there has been a resurgence of interest in the use of mealworms as an alternative source of protein for livestock and aquaculture feed^28,29^.

Aquaculture is the fastest-growing animal feed producing sector that traditionally utilizes fishmeal as its key protein ingredient in the formulation of commercial aquafeeds. Fishmeal has high protein content and balanced essential amino acids (EAA) profile such as methionine (Met), leucine (Leu), and lysine (Lys)^30–32^. Tilapia is one of the most widely and successfully cultured groups of freshwater omnivorous cichlids^29,30^. Tilapias are adaptable, highly resistant to disease, and accept lower-cost diets from terrestrial-based ingredients. Several tropical and sub-tropical countries now culture tilapia in various semi-intensive farming practices^33^. To contend with expansion and intensification, efficient low-cost, and low pollution feeding systems are required. For instance, in 2010, 85% of the global tilapia production was based on the provision of commercial feeds and anticipated to further increase to 95% by 2020^34^.

The successful substitution of fishmeal or soybean meal with insect meal in the diets of rainbow trout (*Oncorhynchus mykiss*), tilapia (*Oreochromis niloticus*), common catfish (*Ameiurus melas*), and Siberian sturgeon (*Acipenser baerii*) have been reported^35–40^. Rainbow trout fed with a diet containing 25% MwM and 50% MwM showed good growth performance^40^. In another study, 50% substitution of fishmeal with MwM brought about poor feed conversion efficiency and a reduction in the growth of European sea bass^38^. In one of our studies, African catfish (*Clarias gariepinus*) fed with 50% to 100% of live mealworms had reduced feed intake (FI), weight gain (WG), and higher body lipid content^35^. The substitution of fishmeal with cricket meal in African catfish (*Clarias gariepinus*)^41^ and black soldier fly (*Hermetia illucens*) in rainbow trout (*Oncorhynchus mykiss*) diet^42–44^ was also reported. Their findings showed improved body weight gain and specific growth rate at 100% and 75% cricket meal and increased richness and diversity in gut microbiota, respectively. The differences in the growth performance observed in various fish feeding trials could be due to variations in the species, stage of development used, rearing conditions, diet, nutritional composition, and digestibility of the feed^45^. Therefore, those factors should be considered before MwM is incorporated into the diet of a fish species.

Mealworm-based meals as an ingredient in fish feed would lessen the growing demand for fishmeal by the aquaculture feed industries worldwide. However, the present practice of breeding mealworms on food-grade (oat, flour, and yeast) or imported substrates (wheat bran) is unsustainable^19^ as feed constitutes about 23% of the mealworm farming^27^. Mealworm meal use in aquafeed would be an attractive option if the insects are of high quality, readily available and if the production costs are lower than that of other sources of protein^46,47^. In the year 2020, the market prices of mealworms were 65–70 USD/kg in South Korea, 12.9–20 USD/kg in Europe, 10.8–14 USD/kg in the United States, and 8.4–9.4 USD/kg in China^48,49^. The relatively higher prices of mealworm meals will hamper their usage in Aquafeed. Furthermore, commercial mealworms accumulate a high amount of lipid (~ 30%) lacking in beneficial omega-3 fatty acids desirable in fish nutrition. High-lipid MwM prevented the addition of fish oil, rapeseed oil^50^, and cod liver oil in a 100% MwM-based African catfish diet (*Clarias gariepinus*) formulation^35^. Low-lipid MwM will result in a more affordable and sustainable source of protein and allow greater control over total dietary lipids in the aquafeed formulation. Although, interesting results have already been reported using defatted insect meals as fishmeal replacement^51–53^. However, the defatting process involves high mechanical energy or large volumes of hazardous solvents. We, therefore, aimed at lowering the lipid and increasing the protein content of the mealworms through the PHA biological recovery process and to simultaneously assess the potential of the mealworms (by-products) in replacing fishmeal fully/partially in tilapia feed formulation.

## Methodology

### Feed preparation

The Bacterial cells were produced via a fermentation process (Refer to our previous publication^17^ for the detailed procedure), freeze-dried to a moisture content of about 5%, pulverised to powder using a laboratory blender (HGB660 Waring blender, USA), passed through a standard 0.5 mm mesh sieve (No. 35, DER SHUENN) and stored at room temperature. The cells contained 54 wt% of PHB and 46% of residual cells. Wheat bran, a conventional mealworm feed (11% moisture content) was obtained from the farm and used as a control diet.

### Mealworm production and feeding

Mealworms (2.5-month-old) were obtained from an insect farm (Yuan Yang Trading, Ipoh, Malaysia). At this larval age, the bacterial cells serve as a finishing diet to produce high-protein, lower-lipid commercial size mealworms (3 months old). This will allow greater control over total dietary lipids when high inclusion levels of mealworm meal are used.

**Figure 1:**
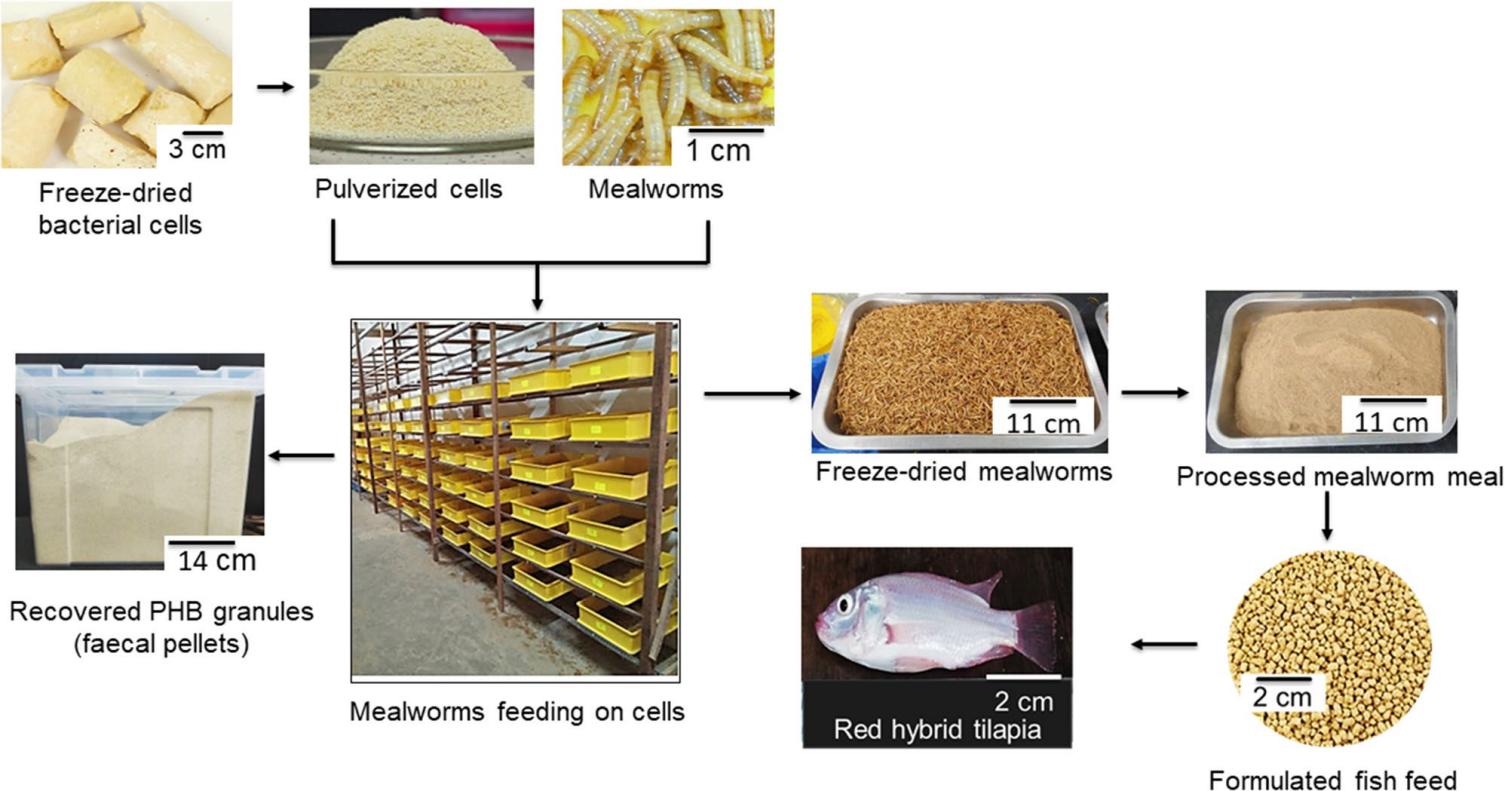
Process flow of the experiment.

An amount of 10 kg (200 g per tray) of the mealworms was placed in fifty plastic trays (50 × 47 × 9 cm) and fed ad libitum a total of 14.96 kg of the freeze-dried cells. Upon complete consumption of the feed, the fresh feed was provided based on the new mealworm weight. The fecal pellets were separated from the mealworms using a 0.5 mm size mesh sieve and weighed. The temperature and relative humidity were maintained at 30 ± 2.0 °C and 72 ± 3.0% RH respectively. The experiment lasted for 16 days because the mealworms (now 3 months old) have reached the market size. Figure 1 shows the process flow of the experiment.

A portion of the same batch of mealworms was fed on wheat bran (control) for 16 days. At the end of the experiment, the mealworms were starved for 24 h to clear their gastrointestinal tract from feed and the feces. Mealworms were kept overnight at − 20 °C, freeze-dried, and pulverized using a laboratory blender (HGB660 Waring Blender, USA) into a homogeneous MwM powder. The proximate (moisture, crude protein, ash, and fiber) were determined^54^ and the nutritional compositions of the MwM were measured before its use (as a protein source) for the replacement of fishmeal in the red hybrid tilapia feed formulation. Total crude lipid content was determined by the chloroform/methanol extraction method^55^.

### Determination of fatty acid

The extracted oil from the mealworms was converted into their fatty acid methyl esters (FAME)^56,57^. An amount of 100 mg of the crude lipid extract and 4 mL of methanolic sodium hydroxide (2% w/v NaOH in methanol) solution were mixed in a 50 mL round bottom flask. About 2–3 glass beads were added, and the mixture refluxed at 75 °C for 10 min. Subsequently, 5 mL of Boron trifluoride (BF3) was added and further heated for 3 min until the complete disappearance of oil globules. About 4 mL of heptane was added, and the mixture was further heated for 1 min. The flask was cooled down at room temperature before the addition of 10 mL of saturated sodium chloride solution. The mixture was swirled several times, and the solution was further added to the 50 mL mark on the flask. The top organic layer was transferred into a test tube containing sodium sulfate to remove traces of water. Samples were lastly transferred into a GC vial, tightened, and stored at − 20 °C. The FAME analysis was carried out using gas chromatography (Model GC- 2010, Shimadzu, Japan) equipped with a flame ionization detector and Shimadzu GC solution integrator. The esters were separated on an SPTM-2560 fused silica capillary column (100 m × 0.25 mm ID, 0.2 μm film thickness, Supelco, Bellefonte, PA, USA). The initial column temperature was 140 °C for 2 min, then increased to 225 °C at 2 °C/min for 5 min. Lastly, to 240 °C at 2 °C/min for 3 min. A split ratio of 1:100 was used for the SPL-2010 injector. Both the injector port and detector temperatures were set at 250 °C. Nitrogen was used as the carrier gas. The resulting peaks were identified by comparing retention time with a known standard (Supelco 37 Component FAME Mix, PUFA No.2).

### Amino acid analysis of bacterial cells and mealworms

About 0.1 to 0.2 g of each sample was hydrolyzed by adding 5 mL of 6 N HCl into the boiling tube and placed in an oven at 110 °C for 24 h. After 24 h, the samples were kept in a desiccator to cool down. Then the 5 mL of hydrolyzed sample was transferred to a 100 mL volumetric flask and mixed with 400 μL of 50 μmol/mL of internal standard (alpha amino butyric acid) and made up to 100 mL with distilled water. The sample was then filtered through a Whatman No. 1 filter paper and then followed by filtering using a syringe filter. Ten microlitres of the sample was mixed with 70 μL of borate buffer and 20 μL of AQC reagent (6-aminoquinolyl-N-hydroxysuccinimdyl carbamite).

The amino acid composition of the cells and mealworms was analysed at Universiti Putra Malaysia (Putra Info port), using the acid hydrolysis method. About 10 μL from the 100 μL sample prepared was injected into HPLC (High Performance Liquid Chromatography) (Waters, Model: 1525, USA) equipped with Binary HPLC Pump, 717 Plus auto-sampler Waters (R) and Waters 2475 Multi λ Fluorescence detector with the wavelength excitation 250 nm and emission 395 nm. BreezeTM software version 3.20 was used to identify, integrate, and quantify the chromatography peaks from the samples and compared with standard (Amino acid standard H, Pierce, Rockford, Illinois, USA).

### Experimental diets

A total of five isonitrogenous (35%), isolipidic (8%), and isocaloric (400 kcal/100 g diets) experimental diets were formulated. Diet formulations were based on the values in the ingredients. Fishmeal was substituted with MwM at grade level of 0%, 25%, 50%, 75% or 100% and labelled as MwM0 (control diet), MwM25, MwM50, MwM75 and MwM100, respectively (Table 1). Mineral and vitamin premix was added to the diets. Corn starch was used to fulfil the energy requirement while carboxymethyl cellulose (CMC) was used as a binding agent. Alpha-cellulose was added as a bulking agent. See Supplementary Table 1 for the detailed diet formulation.

**Table 1:** Ingredients and proximate composition (g/kg dry diet) of experimental diets with increasing substitution level of fishmeal with mealworm meal.

Ingredients	Diet^a^				
	MwM0	MwM25	MwM50	MwM75	MwM100
Fish meal^b^	310.01	230.30	156.00	77.00	0.00
Mealworm meal^c^	0.00	81.20	163.00	244.00	325.00
Soybean meal	200.80	200.80	200.80	200.80	200.80
Fish oil	33.30	32.00	30.50	29.20	27.80
Corn oil	14.00	14.00	14.00	14.00	14.00
Corn starch	245.50	245.50	245.50	245.50	245.50
Vitamin premix^d^	30.00	30.00	30.00	30.00	30.00
Mineral premix^e^	20.00	20.00	20.00	20.00	20.00
CMC	20.00	20.00	20.00	20.00	20.00
Dicalcium phosphate	10.00	10.00	10.00	10.00	10.00
Alpha-cellulose	116.60	113.50	110.20	109.50	106.90
**Proximate composition**					
Crude protein	369	375	378	377	386
Crude lipid	87	87	86	87	84
Dry matter	867	874	860	874	869
Moisture	133	126	140	126	131
Ash	93	86	81	72	65
Crude fiber	5	12	19	27	33
Nitrogen free extract^f^	446	440	436	437	432

*CMC* carboxymethyl cellulose.

^a^MwM denotes a mealworm meal. The number following the letters denotes the % of MwM added, for each diet.

^b^Danish fishmeal purchased from Cargill, Penang, Malaysia (dry matter: 86.9%; crude protein: 79.0%; crude lipid: 8.6%).

^c^Fed with *C. necator* cells as finishing diet (dry matter: 94.2%; crude protein: 75.47%; crude lipid: 9.9%; ash: 6.8%; fiber: 11.0%).

^d^Vitamin premix (g kg^−1^ of premix): inositol, 5; ascorbic acid, 45; choline chloride, 75; niacin, 4.5; pyridoxine HCl, 1; riboflavin, 1; thiamin mononitrate, 0.92; cholecalciferol, 0.083; calcium d-panthothenate, 3; retinyl acetate, 0.6; dl-α-tocopheryl acetate (500 IU g^−1^), 8; menadione sodium bisulfide, 1.67; d-biotin, 0.02; folic acid, 0.09; cellulose, 845.11; vitamin B_12_, 0.00135.

^e^Mineral premix (g kg^−1^ of premix): calcium lactate, 327; Ca(H_2_PO_4_)_2_·H_2_O, 270.98; FeSO_4_·H_2_O, 25; KCl, 50; MgSO_4_·7H_2_O, 132; NaCl, 60; KI, 0.15; MnO_2_, 0.8; CuSO_4_·5H_2_O, 0.785; CoCO_3_, 1; ZnO, 3; CaCO_3_, 129.27; Na_2_SeO_3_·5H_2_O, 0.011.

^f^Nitrogen free extract = 100 − (lipid + crude protein + fiber + ash).

The powdered ingredients were weighed and mixed to homogeneity in a Hobart mixer for 30 min. Fish oil and corn oil were gradually added and blended for 15 min. An amount of 300 mL of distilled water/kg of diet was added to the homogenous mixture to achieve a moist paste. The resulting moist mash was then extruded through a locally assembled meat mincer machine fitted with a 2 mm die. The feed strands were collected on a wire mesh, air-dried overnight at room temperature, and crushed to the desired length.

### Experimental setup, feeding, and sample collection for evaluation of growth and feed conversion efficiency

Red hybrid tilapia, *Oreochromis* sp., fingerlings were purchased from Chia Bee fish farm (Sungai Petani, Kedah, Malaysia) and acclimatized for two weeks on commercial feed (Cargill, Malaysia) in a 1000 L fibreglass tank at the Fish Nutrition Laboratory, Universiti Sains Malaysia.

Approval for this study was obtained from USM Institutional Animal Care and Use Committee, Universiti Sains Malaysia, Approval No: USM/IUCUC/2018/(112) (916). The handling and use of the subject were following the institutional guidelines. All methods are reported in accordance with ARRIVE guidelines.

A total of fifteen 95 L rectangular shaped glass aquaria (70 cm length × 30 cm width × 46 cm height) were set up for the feeding trial. Triplicate groups of 12 fish were randomly placed in each tank. Public utility (sand-filtered) water flowed through the aquaria at a rate of 15 L/h. Airstones were set in each aquarium for continuous aeration. The water pH, temperature, and dissolved oxygen were measured using a portable multi-probe CyberScan PCD 650 water quality meter (EuTech Instruments, Malaysia) and controlled at 7.0 ± 0.2, 29.0 ± 1.0 °C, and 6.0 ± 0.1 mg/L, respectively. The aquaria were housed in a room illuminated with fluorescent lights automatically set for continuous 14 h light per day.

A total of 20 fish were randomly selected from the acclimatization tank, starved for 24 h to clear their digestive tracts, and used for initial whole-body composition analysis. Triplicate groups of the fish were randomly assigned to the experimental diets and hand-fed to apparent satiation twice daily at 0830 and 1630 h. The fish were batch-weighed by tank weekly (see Supplementary Table 2), and the values were used to analyze their growth performance in terms of final body weight, weight gain, and specific growth rate (SGR), while feed conversion ratio (FCR) and protein efficiency ratio (PER) was calculated to evaluate the feed utilization efficiency. The feeding trial was conducted for eight weeks.

### Sample collection and analysis

At the end of the eight weeks feeding trial, 24 h after the last feeding, all fish were counted, individually weighed, and their length was measured. Five fish per tank were randomly selected and anesthetised with 80 mg/L tricaine methanesulphonate, (MS‐222, Sigma, St. Louis, MO, USA) then euthanised at − 20 °C for subsequent whole-body proximate analysis. The remaining fish were dissected to obtain tissue samples such as liver, intestine, and fat. The organs were weighed and the body indices [viscerosomatic index (VSI), hepatosomatic index (HSI), intraperitoneal fat index (IPF), and condition factor (CF)] were calculated^58–60^ as the percentage of the organ to the whole-body weight of individual fish and used to assess the nutritional status of the fish.

### Apparent digestibility coefficient (ADC) and chemical analysis of faeces

The fish were fed for four weeks before faeces collection began. All the aquaria were cleaned before feeding to ensure fresh faeces were collected. Faeces were siphoned after 3 h of feeding and filtered through a fine-mesh net. Only intact faeces strands from each aquarium were collected in Eppendorf tubes, pooled, and kept at − 20 °C. Samples were finely grounded before the proximate composition and apparent digestibility analysis. Feed nutrient digestibility was determined using the acid-insoluble ash (AIA) indicator method (indirect method)^61^. The grounded samples (feed and faeces) were placed in an oven at 103 °C for 24 h and allowed to cool down in a desiccator before weighing. Approximately 1 to 2 g of each sample was weighed in the ash crucible and ashed at 550 °C for 5 h. Ash samples were poured into a 250 mL beaker, 100 mL of 2 N HCl were added, and allowed to boil for 5 min. The hot hydrolysate was filtered using ashless filter paper and then rinsed with hot distilled water. The filter paper with the residue was placed into a pre-weighed ash crucible and ashed at 550 °C for 5 h. Samples were placed in a desiccator to cool and then weighed before proximate composition analysis^62^.

### Statistical analysis

All experimental data were shown as mean ± standard error (*n* = 3) and subjected to one-way analysis of variance (ANOVA) to determine the significant differences among the dietary treatments. Differences among means were further analyzed by using Duncan’s Multiple Range Test and were significant when P-value was < 0.05. The statistical analysis was performed using SPSS statistics 17.0 for Windows (SPSS Inc., Chicago, IL, USA).

## Results

### Nutritional composition, growth performance, and mineral composition of the mealworms

Table 2 shows the feed intake, growth performance, and feed conversion efficiency of mealworms fed with bacterial cells for 16 days. The weight of both cell-fed and wheat bran-fed mealworms increased from 10 to 11.83 kg and 20.5 kg upon consumption of 14.96 kg of cells, and 70.90 kg of wheat bran respectively. About 7.99 kg of a fecal pellet (PHB granules) containing 67 wt% of PHB was recovered.

**Table 2:** Growth performance, feed utilization efficiency, proximate composition (% DM), and mineral composition (mg/100 g on a dry weight basis) of cells, cell-fed and commercial mealworms*.*

Analysis		Mealworms			
		Initial* (74 days old)	Cell-fed	Wheat bran-fed**	
			(90 days old)		
Starting weight (kg)		10.0			
Final weight (kg)			11.83	20.50	
Weight gained (kg)			1.83	10.50	
Diet consumed (kg)			14.96	70.90	
Feces recovered (kg)			7.99	50.58	
FCR			8.20	6.80	
ECI (%)			12.2	26.4	
Weight of freeze-dried mealworms (kg)			2.67	ND	
**Proximate composition (%)**^**a**^					
Moisture		62.8 ± 0.3	77.4 ± 1.1	60.9 ± 0.3	
Crude lipid		26.3 ± 0.2	9.9 ± 0.2	31.7 ± 0.5	
Crude protein		60.6 ± 1.2	75.4 ± 0.5	55.5 ± 0.8	
Ash		4.2 ± 0.1	6.8 ± 0.1	3.5 ± 0.0	
Fiber		9.7 ± 0.1	11.0 ± 0.2	10.2 ± 0.0	
**Mineral composition (mg/100 g)**^**b**^					
Elements	Cells				^62^
Na	185.7	150.5	194.4	103.6	108.8
Ca	23.0	93.2	112.6	71.1	78.4
Fe	2.8	14.2	14.0	10.2	10.0
Mg	51.4	455.4	623.7	329.8	315.2
K	282.4	1251.7	1350.8	1039.6	737
Zn	1.9	9.1	15.3	10.2	11.7
Cu	–	4.3	3.0	2.3	2.0

*FCR* feed conversion ratio on a fresh weight basis, *ECI* feed conversion efficiency on a fresh weight basis, *ND* not determined.

^a^Values are mean of triplicate groups and presented as mean ± SE.

^b^Values were based on a single analysis.

*Mealworms raised on wheat bran.

** Fed by the mealworm farmers.

Values are given as mean ± SD. Superscripts denote significant differences.

Mealworms fed with bacterial cells were of higher protein but lower lipid content compared to wheat bran as shown in Table 2. The moisture (60.9%), protein (55.5%) and ash (3.5%) contents of the mealworms fed with wheat bran were lower compared to those of cell-fed mealworms. The lipid content of cell-fed mealworms decreased from 26.3 to 9.9%, while that of wheat bran-fed mealworms increased from 26.3 to 31.7%.

The mineral composition of bacterial cells and mealworms is shown in Table 2. The cells contained high amounts of potassium (K), sodium (Na), magnesium (Mg), and calcium (Ca) but lacked copper (Cu). The cell-fed mealworms were higher in minerals compared to wheat bran-fed larvae.

### Amino acid profile of the bacterial cells and mealworms

Table 3 shows the essential amino acid (EAA) and non-essential amino acid (NEAA) composition of the bacterial cells and mealworms. The total EAA in the cells was 423 mg/g and the concentrations of arginine and leucine were highest (78 mg/g and 62 mg/g, respectively) while methionine was lowest (18 mg/g). Among the NEAA, glutamic acid concentration was highest (219 mg/g) while tyrosine was the least (22 mg/g). The total concentration of amino acids in the cells was 951 mg/g. The EAA composition was about 50% of the total amino acid content of mealworms. The total EAA and NEAA were the same for both cells and wheat bran-fed mealworms.

**Table 3:** Amino acid profile (g/100 g of protein) of bacterial cells, cell-fed, and wheat bran-fed mealworms. Values were based on a single analysis.

	Amino acids	Cells	Mealworms		Amino acid requirement of tilapia^63^
			Cell-fed	Wheat bran-fed	
EAA	Lysine	5.4	6.6	6.3	5.1
	Leucine	6.2	9.1	7.7	3.4
	Isoleucine	3.9	5.3	5.4	3.1
	Methionine	1.8	2.8	3.3	2.7
	Arginine	7.6	6.5	7.5	4.2
	Valine	4.9	6.2	6.6	2.8
	Threonine	4.4	5.2	5.2	3.8
	Phenylalanine	3.8	5.5	4.4	3.8
	Histidine	4.3	3.6	5.1	1.7
Total EAA		42.3	50.8	51.5	30.6
NEAA	Aspartic acid	6.0	7.7	7.2	
	Serine	4.7	4.9	5.5	
	Glutamic acid	21.9	12.7	10.6	
	Glycine	6.0	6.1	5.9	
	Tyrosine	2.2	3.1	5.3	
	Alanine	5.2	7.3	7.0	
	Proline	6.8	7.3	6.8	
Total NEAA		58.2	49.1	50.5	

*EAA* essential amino acids (tryptophan not determined), *NEAA* non-essential amino acids.

### Fatty acid composition of mealworms

**Figure 2:**
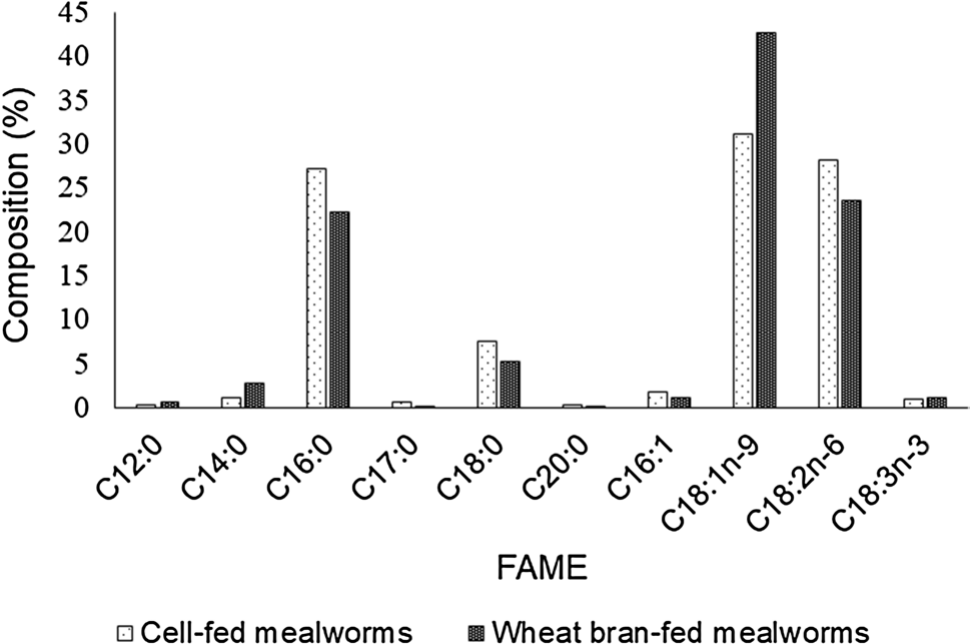
Fatty acid methyl ester composition (%) of the extracted lipid from mealworms fed with bacterial cells and wheat bran.

Figure 2 shows the fatty acid methyl composition of the mealworms. Oleic, linoleic, palmitic, and stearic fatty acids were the main components, comprising about 94% of the total fatty acids. Oleic acid dominated the four fatty acids at a relative percentage of 31% and 42% for the cell-fed and wheat bran-fed mealworms respectively. The second most abundant fatty acids for both mealworms are linoleic acid and palmitic acids at about 28% and 23%, respectively.

### Growth performance and feed utilization efficiency of red hybrid tilapia

The growth performance and feed utilization efficiency are presented in Table 4. At the end of the experiment, fish on MwM0 (control diet) showed significantly (*P* < 0.05) higher final body weight (FBW) of 41.41 g, weight gain (WG) of 787.72%, specific growth rate (SGR) of 4.04%/day, feed intake of 4.77 g and the best FCR of 0.86. For the MwM-based experimental diets, there was no significant difference in FBW and WG among investigated diets up to MwM75. The MwM25 substitution gave the highest SGR (3.56%/day) followed by MwM50 (3.53%), MwM75 (3.32%) and MwM100 (2.95%). As expected, a similar trend was observed with the FI (g/fish) values, which were 4.48, 4.32, 4.19, and 3.58, respectively.

**Table 4:** Growth performances, feed utilization efficiency, Somatic indexes, condition factor and apparent digestibility coefficient (% dry matter) of tilapia fed increasing dietary levels of mealworm meal^1^.

Components	Experimental diets*				
	MwM0	MwM25	MwM50	MwM75	MwM100
IBW^2^ (g)	4.67 ± 0.02	4.67 ± 0.02	4.67 ± 0.02	4.68 ± 0.03	4.69 ± 0.02
FBW^3^ (g)	41.41 ± 2.95^c^	31.93 ± 1.61^b^	31.40 ± 1.61^b^	28.28 ± 3.08^b^	23.09 ± 0.62^a^
WG^4^(%)	787.72 ± 59.5^c^	583.02 ± 34.3^b^	572.3 ± 33.2^b^	503.77 ± 63.2^b^	392.82 ± 15.3^a^
SGR^5^ (%/day)	4.04 ± 0.13^d^	3.56 ± 0.09^c^	3.53 ± 0.09^bc^	3.32 ± 0.19^b^	2.95 ± 0.06^a^
FI^6^(g/fish)	4.77 ± 0.13^d^	4.48 ± 0.19^c^	4.32 ± 0.07^bc^	4.19 ± 0.08^b^	3.58 ± 0.01^a^
FCR^7^	0.86 ± 0.05^a^	0.90 ± 0.04^ab^	0.93 ± 0.01^ab^	1.20 ± 0.32^b^	1.15 ± 0.16^ab^
PER^8^	3.14 ± 0.18^b^	2.97 ± 0.13^b^	2.85 ± 0.04^ab^	2.31 ± 0.55^a^	2.26 ± 0.37^a^
Survival^9^(%)	70.83 ± 12.50^a^	88.9 ± 4.85^b^	86.1 ± 4.85^ab^	77.8 ± 9.58^ab^	86.1 ± 12.73^ab^
HSI^10^	2.03 ± 0.54	1.18 ± 0.17	1.73 ± 0.88	1.16 ± 0.32	1.97 ± 0.63
VSI^11^	2.71 ± 1.01	1.94 ± 0.94	2.53 ± 0.78	2.52 ± 0.58	2.28 ± 1.01
IPF^12^	0.17 ± 0.15	0.13 ± 0.06	0.21 ± 0.10	0.21 ± 0.24	0.14 ± 0.09
CF^13^ (g/cm^3^)	2.25 ± 0.09	2.17 ± 0.02	2.10 ± 0.09	1.96 ± 0.10	1.80 ± 0.03
Dry matter (% ADC)^14^	71.97 ± 0.25^b^	78.67 ± 0.43^c^	74.98 ± 0.52^b^	74.27 ± 0.32^b^	66.4 ± 0.15^a^
Protein (% ADC)	90.96 ± 1.11^c^	91.41 ± 1.31^c^	89.07 ± 0.69^b^	88.55 ± 0.81^b^	83.64 ± 0.09^a^

*MwM denotes mealworm meal. The number following the letters denotes the % of MwM added, for each diet.

^1^Values are the mean ± S.E. of triplicate groups of fish. Values with different superscript letters in each row are significantly different (P < 0.05). The lack of superscript letters indicates no significant differences among treatments.

^2^Initial Body Weight (g).

^3^Final Body Weight (g).

^4^Expressed as the percent of initial body weight at the end of 8 weeks.

^5^Specific growth rate (% day^−1^) = 100 × (ln final − ln initial fish weight)/days.

^6^Feed Intake (g/fish) = total dry feed intake/number of fishes over 54 days.

^7^Feed conversion ratio = total dry diet fed (g)/wet weight gain (g).

^8^Protein efficiency ratio = wet weight gain (g)/total protein intake (g).

^10^Hepatosomatic Index (HSI) = 100 × [liver weight (g)/body weight (g)].

^11^Viscerosomatic index (VSI) = 100 × [viscera weight (g)/body weight (g)].

^12^Intraperitoneal fat index (IPF) = 100 × [intraperitoneal fat weight (g)/body weight (g)].

^13^Condition factor (CF) = [(100 × weight of the fish (g))/(total length of fish (cm)^3^]. ^14^Apparent digestibility coefficient.

### Somatic indexes, condition factor, and apparent digestibility coefficients

There were no significant differences in the somatic indexes, condition factor and haematocrit values of fish fed the various diets (Table 4). The VSI, HSI, IPF, and condition factor results did not differ significantly in red hybrid tilapia fed with different experimental diets.

As shown in Table 4, the inclusion of MwM in the red hybrid tilapia diet had a significant effect on the apparent digestibility coefficients of dry matter (ADCDM) and that of protein (ADCP). The ADCDM and ADCP were highest (78.67% and 91.41%, respectively) in fish fed with MwM25. Fish fed with a control diet, MwM50 or MwM75 showed no significant difference in ADCDM. However, the ADCDM of fish fed with the MwM100 diet was significantly lower at 66.4%. The ADCP was similar for the control diet (90.96%) and MwM25 (91.41%) diets. A gradual decrease in ADCP was recorded from 89.07% in fish fed with MwM50 to 83.64% in fish fed with MwM100.

### Whole-body proximate composition analysis

As shown in Table 5, the whole-body protein and lipid were highest for fish fed with the control diet, MwM75, and MwM100, ranging from 14.52 to 14.59% and 3.71% to 3.97%, respectively. Fish fed MwM25 and MwM50 recorded the lowest whole-body protein (13.9 and 13.74%) and lipid (3.35% and 3.48%) respectively. The whole-body ash was highest in fish fed with MwM75 and MwM100 (4.768 and 4.74%), respectively, followed by those fed with a control diet and MwM50 (4.50% and 4.28%, respectively), and lowest in fish fed with MwM25 (4.04%).

**Table 5:** Whole body composition (% wet weight) of tilapia fed with increasing dietary levels of mealworms.

Diets*	Moisture	Protein	Lipid	Ash
Initial	76.1 ± 0.9	14.56 ± 0.17	4.67 ± 0.13	3.65 ± 0.03
MwM0	76.3 ± 1.4^ab^	14.59 ± 0.22^b^	3.97 ± 0.31^b^	4.50 ± 0.30^ab^
MwM25	78.1 ± 2.0^b^	13.95 ± 0.04^a^	3.35 ± 0.15^a^	4.04 ± 0.14^a^
MwM50	78.0 ± 0.9^b^	13.74 ± 0.21^a^	3.48 ± 0.16^a^	4.28 ± 0.26^ab^
MwM75	75.2 ± 1.4^a^	14.56 ± 0.11^b^	3.96 ± 0.10^b^	4.68 ± 0.16^b^
MwM100	76.2 ± 1.2^ab^	14.52 ± 0.22^b^	3.71 ± 0.29^ab^	4.74 ± 0.31^b^

Values are the mean ± S.E. of triplicate groups of fish. Mean values within the same column with the different superscripts are significantly different (*P* < 0.05).

*See Table 2 footnote for diet description.

## Discussion

The mealworms consumed the freeze-dried cells and excreted the undigested PHB granules as supported by the previous studies carried out on the use of *C. necator* as single-cell proteins^9,13,64^. The weight of wheat bran-fed mealworms was much higher than those supplied with cells because PHA is a storage material of no nutritive value as it is not digested by the mealworms. The FCR (8.2) and efficiency of conversion of ingested feed (ECI) (12.2%) were as earlier reported^17^. Low feed consumption affects growth rate which in turn affects feed conversion to biomass^65^. Interestingly, cell-fed mealworms showed a higher protein and lower lipid content than wheat bran-fed mealworms, due to the higher protein content of *C. necator* H16. According to the literature, insect larvae accumulate a large amount of lipid when fed with high carbohydrate feed^66–68^. For instance, the reported lipid content of the mealworms utilized in feed formulations is between 30% and 42.48%^69–71^. Commercial-sized mealworms (3 months old) are usually high in lipid content (~ 30%). Such mealworms may not be a preferred option to the aquafeed industry due to the need for excessive energy and the additional cost of lipid extraction. For instance, red hybrid tilapia has a dietary lipid requirement from 4% to 8%. For a maximal inclusion of mealworm meal, the mealworms must be defatted to allow for the addition of other desirable oils during diet formulation. The freeze-dried cells are an excellent finishing diet to enhance the protein content and lower the lipid content of market-sized mealworms. Diet did not affect the crude fiber contents of the mealworms and the values agreed with other findings^50,70^.

The cell-fed mealworms were higher in minerals compared to those fed with wheat bran as reported^62^. The mealworms here absorbed substantial amount of minerals from the cells and appeared to be a better source of Ca (112.6 mg/100 g), Fe (14.0 mg/100 g), and Zn (15.3 mg/100 g) than reported^66,72^. According to Makkar and the team, the Ca level of insects is usually lower than that of fishmeal^19^.

Fish have amino acids requirement instead of protein^73^. The quality of a protein is determined by the essential amino acids (EAA) present^52^. The EAA profile of the mealworm meal in this study is similar to that published in a few reports^28,41,63,74^. In fish nutrition, 10 amino acids are considered indispensable: arginine, histidine, isoleucine, leucine, lysine, methionine, phenylalanine, threonine, tryptophan, and valine^75^. The sum of EAA (50.8 g/100 g and 51.5 g/100 g) in the cell and wheat bran-fed mealworms, respectively were higher than that of mealworms raised on soy and maize^76^. The mealworms possess adequate EAA to meet the dietary requirement of tilapia^63^.

The fatty acid results agreed with the previous findings^19,72,77,78^ but were much higher than that reported by Ghosh and the team^62^. About 41%, 27.83%, 16.19%, and 2.32% of oleic acid, linoleic acid, palmitic acid, and myristic acid, respectively were reported^14^. Linoleic acid commonly found in sea products plays a significant role in improving human health^77^. Diet is responsible for the variations in insects’ lipid and fatty acid composition. In comparison, the most abundant FAME reported from BSFL were C12:0 and C14:0 ranging from 0.35–66% to 0.87–13%, respectively^15,79^.

The tilapia growth performance results showed the possibility of up to 75% dietary inclusion of mealworm meal. The MwM100 dietary inclusion negatively affected the growth performance and feed utilization efficiency of the red hybrid tilapia. Kroeckel et al.^80^ also reported decreased fish growth with increasing dietary insect meal resulting from decreased diet acceptability and feed intake. Similarly, Belghit et al.^81^ reported an insignificant difference in fish growth at up to 85% fishmeal replacement. The mealworms from biological recovery gave better results compared to other reports. For instance, most researchers reported an MwM inclusion level of 25–50% without any significant loss in the growth performance of rainbow trout^40^ and European sea bass^38^. Similarly, Xiao et al.^82^ observed the highest growth in *Salmo salar* at 25% fishmeal replacement with black soldier fly meal. Others reported decreased FBW and WG in fish from MwM inclusion levels of 25%^18,81,83^. Khosravi et al.^28^ reported a decreased growth of juvenile rockfish from dietary mealworm inclusion levels above 16%. Ng et al.^35^ reported 17% maximum MwM inclusion in the diet of African catfish without adverse effects on the fish growth performance. The inconsistency in the results regarding the highest recommended levels of mealworm meal inclusion as an alternative source of protein in practical fish diets could be due to the differences in mealworm meal composition, diet formulation, culture condition, and fish species. The FCR values were similar for all treatments, however, the substitution of fishmeal with MwM higher than 50% affected the feed utilization efficiency (FI and PER). A similar trend was reported by Kroeckel et al.^80^. Furthermore, the fish fed with MwM25 was comparable to those fed with a control diet in terms of protein efficiency ratio (PER), at 2.97 and 3.14, respectively. The lowest values (2.31 and 2.26) were recorded for MwM75 and MwM100, respectively.

The condition factor is indicative of the feeding condition of the fish, known to increase in well-fed fish^84^. The cell-fed mealworms contain no known antinutritional factors that may affect the body and blood indices of the fish.

The reduction in protein digestibility, fish growth performance, and nutrient utilization with increasing insect meal inclusion agreed with a study^39^. A researcher reported lower ADCP and ADCDM from black soldier fly-based diets^83^. This may be due to less protein concentration in the diet as the concentrations of dietary protein were measured based on the calculated total nitrogen of the feed ingredients used. High levels of chitin may decrease nutrient digestibility and lower fish growth performances^19,84^. However, in other related research, the protein digestibility and dry matter were not affected with black soldier fly meal inclusion in the diets of Atlantic salmon and rainbow trout^42,81^. The variations in results could be attributed to the high variability in species, quality of available market insect meal, and the chitin content of the meal. For example, the digestibility of amino acids from a house fly meal as a broiler diet was over 90% while the digestibility of the crude protein was much lower^85^. In insects, the most common form of fiber is chitin, which may decrease the insect’s crude protein digestibility. The exoskeleton of a mealworm is made up of chitin and *N*-acetylglucosamine, all containing nitrogen atoms that are quantified as protein using the conversion factor (6.25) for conventional food. According to a report^86^, the protein content of the insects is overestimated by the Kjeldahl method, which measures the nitrogen content of a sample. Other nitrogen-containing compounds such as amines, organic compounds, and nucleic acids are quantified as protein by this method^87^. Besides, not all insects’ proteins are digestible for animals. Also, proteins associated with the cuticular matrix may not be accessible although the estimated chitin‐bound N in mealworms was approximately 5.6% of the total N. This value might become more significant with increasing levels of MwM in the diet. The crude fiber measured in this study included chitin, lignin, cellulose, and hemicellulose. Mealworms used here had a crude fiber of 11.0% (dry weight basis), which represents the chitin content. As shown in Table 1, dietary fiber presumed to be mainly chitin increases with an increasing level of MwM in the diet. According to Kroeckel^80^, high incorporation of insect meal in aquafeed decreases feed intake, lowers palatability, digestibility, and nutrient availability, due to increasing chitin content. Similarly, Shiau et al.^32^ recorded reduced growth performance and feed utilization efficiency in tilapia fed with diets containing ˂ 2% of chitin. Although chitinase activity is known to occur in some fish species, it is believed that the complex matrix of insect chitin is responsible for its poor digestion in fish. The chances of contact between chitinases or proteinases with chitin may be reduced due to structural complexity. Consequently, this prevents protein and lipid absorption by the fish gut.

The similarity in nutrient composition of the fish from this study is a notable achievement, as numerous studies reported marked changes in fish whole-body composition at a certain level of dietary insect meal inclusion. According to Ng et al.^35^, dietary mealworm meal inclusion caused a significant increase in the whole-body fat content of African catfish. Belforti and the team^36^ recorded increased protein but decreased dry matter and ash content in the muscle of rainbow trout fed with a diet containing 50% of mealworm meal. From another study, mealworm meal dietary inclusion below 50% did not affect the whole-body proximate composition of European sea bass^38^. In a study involving black soldier fly, a consistent protein but decreasing dry matter and lipid content in juvenile turbot was recorded at 76% of the diet inclusion^80^. The results of this study indicated that the mealworms used for the PHB biological recovery process had no negative effects on the fish bodies and organs.

## Conclusion

We have successfully demonstrated for the first time the possibility of using the high-protein (79%), low-lipid (8.3%) mealworms obtained from biological recovery of PHB from freeze-dried *C. necator* H16 as an alternative insect-based ingredient for red hybrid tilapia feed formulation. Cell-fed mealworm meals are advantageous because they do not require lipid extraction and allow the addition of other types of important dietary oils (corn and fish oil) during the feed formulation. This process has the potential to be included as part of the PHA and mealworm industrial production. Incredibly, a large quantity of PHA could be recovered for various applications. Much optimization is required to ensure that the biological recovery process contributes to a sustainable industrial symbiosis. In addition, larger-scale feeding over an extended period is necessary to obtain more reliable data. Although the growth performance and feed conversion efficiency of the fish on MwM-based diets were lower than those on fishmeal, we believe better performance can be obtained if the diets were formulated based on a lower percentage of total dietary protein contributed by fishmeal to the fish feeds. Chitin tends to decrease the insect’s crude protein digestibility, the feed formulation should be based on a digestible protein to further elucidate the nutritive value of the mealworms as a protein source.

## Supplementary Information

Supplementary Table 1.

Supplementary Table 2.
